# Trends and inequalities in breastfeeding continuation from 1 to 6 weeks: findings from six population-based British cohorts, 1985–2010

**DOI:** 10.1038/s41430-021-01031-z

**Published:** 2021-11-12

**Authors:** Deon A. Simpson, Claire Carson, Jennifer J. Kurinczuk, Maria A. Quigley

**Affiliations:** National Perinatal Epidemiology Unit, Nuffield Department of Population Health, University of Oxford, Oxford, UK

**Keywords:** Epidemiology, Epidemiology

## Abstract

**Background:**

Understanding inequalities in breastfeeding practices may help to explain the UK’s persistently low breastfeeding rates. A recent study using the quinquennial UK Infant Feeding Surveys (IFS) found that sociodemographic inequalities in breastfeeding *initiation* persisted between 1985 and 2010. The present study investigates the sociodemographic inequalities in breastfeeding *continuation* at 6 weeks after birth among mothers who initiated and maintained breastfeeding at 1 week in 1985–2010.

**Methods:**

Data were drawn from the 1985 to 2010 IFS and restricted to mothers who were breastfeeding at 1 week after birth. Time trends in the proportion of mothers in each sociodemographic group were examined. Logistic regression was used to estimate associations between breastfeeding at 6 weeks and sociodemographic factors, adjusting for confounders. Heterogeneity test was used to assess changes in these associations over time.

**Results:**

Sociodemographic inequalities in breastfeeding continuation at 6 weeks persisted over the 25-year period. In most survey years, mothers were most likely to breastfeed at 6 weeks if they were 30 or older versus under 25 (OR 1.49–1.99 across survey years, *I*^2 ^= 0%, heterogeneity *P* = 0.45); completed full-time education over age 18 compared to 18 or younger (OR 1.56-2.51, *I*^2 ^= 58.7%, *P* = 0.03); or of Black, Asian, Mixed, or other ethnicity compared to White (OR 1.45–2.48, *I*^2 ^= 44.8%, *P* = 0.16).

**Conclusions:**

Among mothers breastfeeding at 1 week, those who were younger, White or had fewer years of full-time education were at greatest risk of discontinuing before 6 weeks. This risk persisted over time and was independent of their high risk of not initiating breastfeeding.

## Introduction

The public health and economic significance of breastfeeding are well documented [1, 2]. Despite such compelling evidence, few mothers practise the global recommendations of exclusively breastfeeding their baby for the first 6 months of life and continuing any breastfeeding for at least the first 2 years [3]. Moreover, breastfeeding continuation is shorter in higher-income countries such as the USA and the UK than in resource-limited countries [2]. While over 80% of mothers in these countries initiate breastfeeding, 60% of mothers in the USA ceased breastfeeding exclusively within the first three months after birth in 2018 [4]. In England in 2019, 54% of all mothers ceased any breastfeeding between 6 and 8 weeks after birth [5].

Such low population-based breastfeeding rates often mask even lower breastfeeding practices among subgroups of mothers [6]. Several studies from higher-income countries, including the UK, found significantly lower breastfeeding continuation rates among mothers who are younger, unmarried, or from more disadvantaged socioeconomic groups, mothers with lower educational attainment [7–12], and in the UK, mothers of White ethnicity [13].

In 2019, the authors of this present study presented a trend analysis using data from the UK Infant Feeding Surveys (IFS) and found similar sociodemographic inequalities in the practise of breastfeeding initiation in every survey year from 1985 to 2010 [6]. These inequalities did not change significantly over the 25-year period, and the improvement over time in overall breastfeeding initiation at the population level was driven largely by the decreasing prevalence in the childbearing population of the aforementioned subgroups [6].

This present study investigates whether similar sociodemographic inequalities in the practise of breastfeeding persisted at 6 weeks after birth between subgroups of mothers who initiated and continued any breastfeeding at 1 week after birth in survey years 1985–2010. For each year, we estimate the associations between breastfeeding continuation and sociodemographic factors (adjusted for a range of confounders), and use statistical methods to analyse changes in these associations over time taking into account differences in survey year populations. Few other studies have investigated the trends in breastfeeding continuation in higher-income countries [14–17], and to our knowledge, no study used this meta-analytic approach to investigate trends in sociodemographic inequalities in breastfeeding continuation in the UK over such a long period.

Six weeks represent a critical stage in the postnatal period when breastfeeding practice among UK mothers declines markedly. The presence of trends in inequalities in breastfeeding continuation at 6 weeks can help to predict which women might be more likely to discontinue breastfeeding early. This can inform anticipatory planning and prioritisation of breastfeeding support strategies and responses for the most at-risk women.

## Methods

### Data

This study used data from Stage 1 of the 1985–2010 IFS. For each survey year, samples of mothers were selected over three months from all registered live births in England, Wales and Scotland (Great Britain, [GB]) and Northern Ireland. The initial sample sizes ranged from 8154 to 30,760 mothers in 1985 to 2010, respectively, and included an oversample of births from the lowest socioeconomic group. The IFS questionnaires were mailed in three stages and staggered so that mothers received the Stage 1 questionnaire when their infant was 4–10 weeks old. The data were weighted to correct for differential sampling in the four countries, oversampling of births from the lowest socioeconomic group and survey non-response. Further details are available elsewhere [18].

### Study populations

This study excluded mothers from Northern Ireland because the IFS did not collect data from this country prior to 1990 and ethnicity was not collected. Mothers of multiple births and those who gave birth outside a hospital or maternity unit were excluded due to small numbers. Mothers of babies younger than 6 weeks at Stage 1 were also excluded. In extending our recent trend analysis [6], this present study sought to isolate the factors associated with continuing any breastfeeding at 6 weeks from those associated with initiating and then maintaining any breastfeeding at 1 week—the period showing the sharpest drop-off in breastfeeding each survey year. Therefore, the study populations analysed were further restricted to mothers who were still breastfeeding at 1 week [19]. Following these exclusions, the final populations included in this study ranged from 3552 to 7396 mothers in 1985 to 2010, respectively who gave birth to a singleton in hospital in GB who was at least 6 weeks old at Stage 1 and still being breastfed at 1 week after birth.

### Study outcome

Breastfeeding continuation from 1 to 6 weeks after birth (hereinafter breastfeeding at 6 weeks) was assessed using two questions: (1) “Thinking about the milk that your baby has received over the last 7 days, has he/she had only breast milk, only infant formula or both?”; (2) “How old was your baby when he/she was last given breast milk or put to your breast?” Breastfeeding at 6 weeks was derived by counting all mothers who were still breastfeeding, plus mothers who last gave breast milk or breastfed when their baby was 6 weeks or older.

### Sociodemographic factors

Six maternal sociodemographic factors were defined using data available in each survey year: (1) age when mothers gave birth (hereinafter, age when giving birth); (2) education–age when full-time education was completed; (3) socioeconomic status, using partner’s occupation under the Standard Occupational Classification (SOC) in 1985 to 1995, and mothers’ own occupation under the National Statistics Socioeconomic Classification (NS-SEC) in 2000 to 2010; (4) employment status at 6 weeks after birth; (5) ethnicity; (6) partnership status. Table 1 shows the categories of these sociodemographic factors.

**Table 1:** Distribution of sociodemographic factors in the study population in each survey year.

	Survey years											
	1985		1990		1995		2000		2005		2010	
Population (a)	3552		3633		3178		2792		4768		7396	
Age when giving birth	***N*** **(b)**	**% (c)**	***N***	**%**	***N***	**%**	***N***	**%**	***N***	**%**	***N***	**%**
Under 20	193	(4.8)	143	(3.4)	111	(3.3)	108	(4)	152	(4)	137	(3)
20–24	914	(25.3)	739	(19.6)	461	(14.1)	367	(13.4)	640	(15.2)	792	(13.9)
25–29	1350	(38.3)	1410	(38.9)	1082	(34.7)	748	(26.6)	1143	(26.3)	2027	(28.5)
30 or over	1086	(31.6)	1331	(38.1)	1519	(47.9)	1560	(56.1)	2818	(54.5)	4413	(54.5)
*Missing*	*9*	*0.3*	*10*	*0.3*	*5*	*0.2*	*9*	*0.3*	*15*	*0.3*	*27*	*0.4*
Education—age when completed full-time education												
16 or under *(Least number of years of full-time education)*	1676	(47.1)	1448	(40.3)	982	(31.8)	707	(25.6)	886	(19.2)	872	(13.3)
17 or 18	1099	(31.6)	1303	(36.8)	1178	(38)	913	(33.9)	1451	(31.5)	1745	(25.4)
Over 18 *(Higher education)*	755	(21.4)	853	(23)	994	(30.2)	1156	(40.4)	2392	(49.4)	4702	(61.3)
*Missing*	*22*	*0.6*	*29*	*0.8*	*24*	*0.8*	*16*	*0.6*	*39*	*0.8*	*77*	*1.0*
Socioeconomic status based on partner’s occupation (SOC)												
Managerial and professional	301	(8.9)	392	(11.3)	331	(9.8)	-	-	-	-	-	-
Intermediate	876	(26.5)	875	(25.9)	941	(31.8)	-	-	-	-	-	-
Skilled, Non-manual	344	(10.1)	315	(9.3)	260	(9)	-	-	-	-	-	-
Skilled, Manual	1018	(30.2)	923	(27.2)	670	(22.9)	-	-	-	-	-	-
Semi-skilled	414	(12.2)	385	(10.7)	270	(8.7)	-	-	-	-	-	-
Unskilled	172	(3.2)	70	(1.3)	109	(2.4)	-	-	-	-	-	-
Unclassified	409	(9.1)	655	(14.2)	578	(15.4)	-	-	-	-	-	-
*Missing*	*18*	*0.5*	*18*	*0.5*	*19*	*0.6*	-	-	-	-	-	-
Socioeconomic status based on mother’s occupation (NS-SEC)												
Managerial and professional	-	-	-	-	-	-	1059.0	(37.2)	2187.0	(44.3)	3315.0	(40.4)
Intermediate	-	-	-	-	-	-	590.0	(22.3)	973.0	(20.1)	1402.0	(19)
Routine and manual	-	-	-	-	-	-	579.0	(20.4)	1172.0	(25.4)	1624.0	(23.5)
Never worked	-	-	-	-	-	-	300.0	(11.1)	282.0	(7)	515.0	(9.3)
Unclassified	-	-	-	-	-	-	264.0	(9)	154.0	(3.2)	540.0	(7.9)
Employment status at 6 weeks after birth												
Paid maternity leave	240	(7.2)	880	(25.7)	1254	(40.9)	1317	(46.5)	2834	(58.3)	4597	(59.4)
Unpaid maternity leave	311	(8.9)	241	(6.4)	219	(6.5)	123	(4.1)	169	(3.6)	301	(4.2)
Doing paid work	186	(5.4)	257	(7.7)	225	(6.5)	172	(6.2)	139	(2.4)	394	(5.1)
Not doing paid work	2797	(78.5)	2245	(60.2)	1470	(46.1)	1172	(43.3)	1601	(35.7)	2036	(31.3)
*Missing*	*18*	*0.5*	*10*	*0.3*	*10*	*0.3*	*8*	*0.3*	*25*	*0.5*	*68*	*0.9*
Ethnicity												
White	-	*-*	-	*-*	-	*-*	6,747	(93.1)	3904	(80.6)	5976	(79.6)
Black, Asian, Mixed, Multiple or other	-	*-*	-	*-*	-	*-*	415	(6.9)	813	(19.4)	1,131	(20.4)
*Missing*	*-*	*-*	*-*	*-*	*-*	*-*	*206*	*2.8*	*51*	*1.1*	*289*	*3.9*
Partnership status												
Married	3020	(87.5)	2859	(81.2)	2,315	(74)	1824	(66.6)	2994	(64.7)	4937	(64.8)
Living with a partner	240	(6.6)	383	(10.6)	494	(16.2)	620	(22.7)	1097	(23.6)	1773	(25)
Single	273	(5.9)	373	(8.2)	350	(9.8)	329	(10.6)	603	(11.8)	613	(10.2)
*Missing*	*19*	*0.5*	*18*	*0.5*	*19*	*0.6*	*19*	*0.7*	*74*	*1.6*	*73*	*1.0*

(a) Total unweighted population of mothers analysed for breastfeeding at 6 weeks in each survey.

(b) Number of mothers in each sociodemographic sub-group category, unweighted.

(c) Weighted proportion of mothers in each sociodemographic sub-group category.

Dash (-) = factor not assessed by the IFS in that survey year.

### Statistical analyses

Data from each survey year were analysed separately instead of in a pooled dataset because each survey included its own weights. The proportion of mothers breastfeeding at 6 weeks was estimated for each survey and a test for linear trend was performed.

Multivariable logistic regression was used to estimate independent associations between breastfeeding at 6 weeks and each sociodemographic factor in a survey year. Sociodemographic factors that were associated with breastfeeding at 6 weeks in univariable analysis (*p* < 0.10) were included in multivariable models. Odds ratios (ORs) for the sociodemographic characteristics were adjusted for each other and other factors that were associated with breastfeeding at 6 weeks in univariable analysis including birth- and health-related, direct previous experience of breastfeeding, indirect breastfeeding experience, support with breastfeeding in hospital and home, whether mothers were exposed to breast milk substitutes, and the presence of measures to protect breastfeeding in public (see Supplementary Table A). Multivariable models were fitted in stages and a more conservative *p* value (*p* < 0.05) determined which factors remained in final models in each survey year.

Random-effects meta-analysis models were used to pool the data across survey years under the assumption that there was some variability in associations between survey years [20]. A test for statistical heterogeneity and the *I*^2^ statistic were used to assess if the independent association between each sociodemographic factor and breastfeeding at 6 weeks changed over time [21].

All proportions and ORs were weighted to account for design effects and attrition using survey commands in Stata 13.1 [22]. The IFS had ethical approval and this study required no further approvals as all data are anonymised and publicly available from the UK Data Archive.

## Results

Table 1 shows the changing distribution of the sociodemographic factors among women who were still breastfeeding at 1 week. From 1985 to 2010, there were marked increases in the proportions of mothers who were 30 or over when giving birth, had higher education (completed full-time education over 18), held managerial and professional occupations, and accessed paid maternity leave at 6 weeks after birth. Over the same period, the proportion of married mothers declined while lone parenting and cohabiting relationships increased. The proportion of mothers of Black, Asian, Mixed, or other non-White ethnic groups tripled from 2000 to 2010.

Table 2 shows that the proportion of mothers who breastfed at 1 week and continued breastfeeding at 6 weeks increased steadily across the surveys from 70.1% in 1985 to 79.9% in 2010 (average increase of 2% every 5 years; *p* < 0.001 for linear trend). This table also summarises the univariable analysis results, highlighting that breastfeeding at 6 weeks was associated with each sociodemographic factor before adjustments for confounding (*p* < 0.10).

**Table 2:** Associations between breastfeeding at 6 weeks and maternal sociodemographic factors, Great Britain, 1985–2010: univariable analysis results.

	Survey years											
	1985		1990		1995		2000		2005		2010	
Population analysed (*n*)	3552		3633		3178		2792		4768		7396	
Numbers breastfeeding at 6 weeks (a)	2459		2617		2340		2112		3705		5922	
% of *n* breastfeeding at 6 weeks (b)	70.1		72.8		74.0		75.5		77.6		79.9	
	**OR (c)**	**95% CI (d)**	**OR**	**95% CI**	**OR**	**95% CI**	**OR**	**95% CI**	**OR**	**95% CI**	**OR**	**95% CI**
Age when giving birth												
Under 20	**0.20**	**0.14–0.29**	**0.17**	**0.11–0.25**	**0.22**	**0.15–0.35**	**0.33**	**0.23–0.49**	**0.27**	**0.18–0.41**	**0.39**	**0.25–0.61**
20–24	**0.36**	**0.29–0.44**	**0.37**	**0.29–0.45**	**0.34**	**0.26–0.43**	**0.43**	**0.34–0.55**	**0.47**	**0.38–0.59**	**0.38**	**0.31–0.47**
25–29	**0.36**	**0.53–0.79**	**0.64**	**0.52–0.76**	**0.50**	**0.40–0.61**	**0.59**	**0.48–0.71**	**0.75**	**0.61–0.91**	**0.61**	**0.53–0.72**
30 or over	1.00		1.00		1.00		1.00		1.00		1.00	
Education—age when completed full-time education												
16 or under *(Least number of years of full-time education)*	**0.60**	**0.50–0.71**	**0.66**	**0.55–0.79**	**0.66**	**0.54–0.81**	**0.69**	**0.57–0.85**	**0.72**	**0.58–0.89**	0.93	0.75–1.14
17 or 18	1.00		1.00		1.00		1.00		1.00		1.00	
Over 18 *(Higher education)*	**2.19**	**1.69–2.84**	**2.15**	**1.67–2.77**	**2.74**	**2.13–3.52**	**2.38**	**1.93–2.94**	**2.06**	**1.70–2.49**	**2.25**	**1.92–2.63**
SOC												
Managerial and professional	**3.07**	**2.11–4.46**	**2.17**	**1.56–3.02**	**3.20**	**2.13–4.79**	-	-	-	-	-	-
Intermediate	**1.98**	**1.58–2.47**	**1.73**	**1.36–2.18**	**2.05**	**1.59–2.64**	-	-	-	-	-	-
Skilled, Non-manual	1.12	0.84–1.48	1.33	0.97–1.82	1.24	0.88–1.75	-	-	-	-	-	-
Skilled, Manual	1.00		1.00		1.00		-	-	-	-	-	-
Semi-skilled	0.81	0.63–1.05	**0.74**	**0.56–0.97**	1.03	0.73–1.45	-	-	-	-	-	-
Unskilled	**0.60**	**0.42–0.86**	0.86	0.49–1.53	0.72	0.44–1.19	-	-	-	-	-	-
Unclassified	**0.72**	**0.55–0.92**	0.82	0.65–1.03	0.87	0.67–1.13	-	-	-	-	-	-
NS-SEC												
Managerial and professional	-	-	-	-	-	-	**2.75**	**2.20–3.45**	**2.13**	**1.75–2.59**	**1.89**	**1.59–2.24**
Intermediate	-	-	-	-	-	-	**1.68**	**1.32–2.14**	**1.38**	**1.10–1.73**	**1.24**	**1.02–1.51**
Routine and manual	-	-	-	-	-	-	1.00		1.00		1.00	
Never worked	-	-	-	-	-	-	**1.78**	**1.33–2.39**	**2.40**	**1.62–3.56**	**2.22**	**1.61–3.05**
Unclassified	-	-	-	-	-	-	**1.81**	**1.31–2.49**	**2.91**	**1.69–4.99**	**1.68**	**1.26–2.24**
Employment status at 6 weeks after birth												
Paid maternity leave	1.20	0.87–1.65	**1.24**	**1.02–1.51**	1.10	0.92–1.31	**1.27**	**1.07–1.52**	0.91	0.76–1.08	0.86	0.73–1.01
Unpaid maternity leave	1.12	0.85–1.49	1.38	0.97–1.96	**1.66**	**1.13–2.45**	**1.79**	**1.11–2.87**	1.09	0.68–1.77	0.84	0.58–1.20
Doing paid work	0.81	0.58–1.13	0.98	0.72–1.33	1.06	0.75–1.50	0.81	0.58–1.13	0.96	0.58–1.60	0.92	0.67–1.27
Not doing paid work	1.00		1.00		1.00		1.00		1.00		1.00	
Ethnicity												
White	-	-	-	-	-	-	1.00		1.00		1.00	
Black, Asian, Mixed, Multiple or other	-	-	-	-	-	-	**1.75**	**1.29–2.37**	**2.69**	**2.08–3.48**	**2.74**	**2.18–3.45**
Partnership status												
Married	1.00		1.00		1.00		1.00		1.00		1.00	
Living with a partner	**0.47**	**0.35–0.63**	**0.63**	**0.49–0.81**	**0.65**	**0.51–0.82**	**0.60**	**0.49–0.74**	**0.51**	**0.42–0.61**	**0.53**	**0.45–0.62**
Single	**0.44**	**0.34–0.58**	**0.46**	**0.36–0.59**	**0.52**	**0.40–0.67**	**0.41**	**0.32–0.52**	**0.50**	**0.40–0.64**	**0.44**	**0.36–0.56**

(a) Number of mothers from population of mothers analysed who were breastfeeding at 6 weeks in each survey, unweighted.

(b) Weighted proportion of mothers from population analysed who were breastfeeding at 6 weeks in each survey year.

(c) Odds ratio unadjusted for other factors.

(d) CI: confidence interval.

Bold figures are statistically significant.

*p* ≤ 0.10 unless indicated by asterisks.

Dash (-) = factor not assessed by the IFS in that survey year.

Table 3 shows the multivariable analysis results after controlling for maternal sociodemographic, birth, health, breastfeeding experience and support factors. Breastfeeding at 6 weeks was independently associated with five of the six sociodemographic factors in at least one survey year (*p* < 0.05). Specifically, mothers with higher education were most likely to breastfeed at 6 weeks in every survey year, with adjusted OR (aOR) ranging between 1.41 (95% CI: 1.04–1.91) and 2.15 (95% CI: 1.63–2.84). In most survey years, mothers aged 30 and older were more likely to breastfeed at 6 weeks than younger mothers. Mothers of Black, Asian, Mixed or other ethnic origin were more than twice as likely as White mothers to breastfeed at 6 weeks in 2005 (aOR 2.48, 95% CI: 1.80–3.43) and 2010 (aOR 2.38, 95% CI: 1.81–3.14). Associations between breastfeeding at 6 weeks and socioeconomic status and partnership status were less consistent across the study period. There was no independent association between breastfeeding at 6 weeks and employment status at 6 weeks in any survey year (Table 3).

**Table 3:** Associations between breastfeeding at 6 weeks and maternal sociodemographic factors, Great Britain, 1985–2010: multivariable analysis results.

	Survey years											
	1985		1990		1995		2000		2005		2010	
Population analysed (*n*)	3552		3633		3178		2792		4768		7396	
Numbers breastfeeding at 6 weeks	2459		2617		2340		2112		3705		5922	
% of *n* breastfeeding at 6 weeks	70.1		72.8		74.0		75.5		77.6		79.9	
	**(a) OR**	**95% CI**	**OR**	**95% CI**	**OR**	**95% CI**	**OR**	**95% CI**	**OR**	**95% CI**	**OR**	**95% CI**
Age when giving birth												
Under 20	**0.35**	**0.23–0.55**	**0.26**	**0.17–0.41**	**0.46**	**0.28–0.76**			**0.60**	**0.38–0.97**	0.87	0.49–1.54
20–24	**0.55**	**0.42–0.72**	**0.55**	**0.42–0.72**	**0.55**	**0.41–0.75**			**0.66**	**0.49–0.89**	**0.64**	**0.49–0.83**
25–29	**0.75**	**0.58–0.95**	**0.78**	**0.62–0.99**	**0.71**	**0.56–0.89**			0.84	0.66–1.06	**0.71**	**0.59–0.86**
30 or over	1.00		1.00		1.00				1.00		1.00	
Education—age when completed full–-time education												
16 or under *(Least number of years of full-time education)*	**0.68**	**0.55–0.83**	**0.78**	**0.63–0.96**	**0.77**	**0.62–0.97**	0.88	0.69–1.12	0.85	0.66–1.10	1.11	0.85–1.44
17 or 18	1.00		1.00		1.00		1.00		1.00		1.00	
Over 18 *(Higher education)*	**1.41**	**1.04–1.91**	**1.45**	**1.09–1.93**	**2.15**	**1.63–2.84**	**1.90**	**1.48–2.44**	**1.47**	**1.18–1.85**	**1.66**	**1.37–2.01**
SOC												
Managerial and professional	**1.64**	**1.07–2.50**					-	-	-	-	-	-
Intermediate	1.28	0.98–1.67					-	-	-	-	-	-
Skilled, Non-manual	0.92	0.67–1.26					-	-	-	-	-	-
Skilled, Manual	1.00						-	-	-	-	-	-
Semi-skilled	0.88	0.66–1.17					-	-	-	-	-	-
Unskilled	**0.56**	**0.38-0.84**					-	-	-	-	-	-
Unclassified	0.82	0.59–1.13					-	-	-	-	-	-
NS-SEC												
Managerial and professional	-	-	-	-	-	-	**1.83**	**1.38–2.41**				
Intermediate	-	-	-	-	-	-	**1.34**	**1.01–1.78**				
Routine and manual	-	-	-	-	-	-	1.00					
Never worked	-	-	-	-	-	-	**1.91**	**1.30–2.81**				
Unclassified	-	-	-	-	-	-	**1.47**	**1.00–2.14**				
Employment status at 6 weeks after birth												
Paid maternity leave												
Unpaid maternity leave												
Doing paid work												
Not doing paid work												
Ethnicity												
White	-	-	-	-	-	-			1.00		1.00	
Black, Asian, Mixed, Multiple or other	-	-	-	-	-	-			**2.48**	**1.80–3.43**	**2.38**	**1.81–3.14**
Partnership status												
Married											1.00	
Living with a partner											**0.79**	**0.65–0.95**
Single											**0.67**	**0.50–0.91**

(a) Odds ratio adjusted for maternal (1) maternal sociodemographic characteristics; (2) birth- and health-related factors; (3) mothers’ previous breastfeeding experiences; (4) breastfeeding support in hospital; (5) breastfeeding support at home.

Bold figures are statistically significant.

*p* ≤ 0.05 unless indicated by asterisks.

Dash (-) = factor not assessed by the IFS in that survey year.

**Fig. 1:**
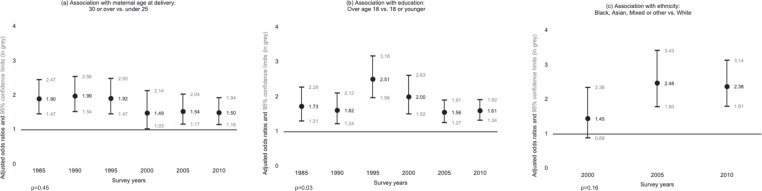
**Trends in the associations between breastfeeding at 6 weeks and maternal sociodemographic factors, Great Britain, 1985 to 2010.** **a** Maternal age when giving birth; **b** Maternal age when completed full-time education; **c** Ethnicity.

Figure 1 shows the trends over time from 1985 to 2010 in the aORs between breastfeeding at 6 weeks and the three most strongly associated sociodemographic factors. There was no evidence of heterogeneity for the effect of age, with mothers consistently being more likely to breastfeed at 6 weeks if they were aged 30 or over compared to under 25 (aOR 1.49–1.99 across survey years, heterogeneity *p* = 0.45; *I*^2^ = 0%). There was some evidence of moderate heterogeneity for the effects of education (heterogeneity *p* = 0.03, *I*^2^ = 58.7%) and ethnicity (heterogeneity *p* = 0.16, *I*^2^ = 44.8%); however, the direction of effect was consistent over the survey years. Specifically, mothers were consistently more likely to breastfeed at 6 weeks if they completed full-time education over 18 versus 18 or younger (aOR 1.56–2.51) or were of Black, Asian, Mixed, or other ethnicity compared to White (aOR 1.45–2.48).

## Discussion

The study findings indicate a steadily increasing trend in the overall rate of breastfeeding continuation from 1 to 6 weeks from 70% in 1985 to 80% in 2010. Despite this trend, there was evidence of sociodemographic inequalities in breastfeeding between subgroups of mothers in a given survey year. Specifically, mothers who were breastfeeding at 1 week were consistently more likely to breastfeed at 6 weeks if they had higher education, were age 30 or older, or of Black, Asian, Mixed or other ethnicities. The observed inequalities likely persisted over the 25 years as there was no strong evidence of consistent changes in these associations over time that might account for the steadily increasing trend in breastfeeding at 6 weeks. Other sociodemographic factors in this present study—maternal socioeconomic status, partnership status and employment status at 6 weeks after giving birth—were not consistently associated with breastfeeding continuation from 1 to 6 weeks after adjusting for confounders.

These observed inequalities in breastfeeding at 6 weeks are similar to those highlighted in our previous trend analysis, which found that younger and White mothers and those with fewer years of full-time education were persistently less likely to initiate breastfeeding over the same 25-year period [6]. All the mothers in this present study initiated breastfeeding. Therefore, the current findings also indicate that even when these subgroups of mothers initiate breastfeeding and maintain breastfeeding at 1 week, they are at significant risk of discontinuing breastfeeding by 6 weeks. This underscores the significant persistent influence of the wider sociocultural sphere in which mothers exist and must manoeuvre to successfully initiate and continue breastfeeding.

Several mechanisms might contribute to the observed sociodemographic inequalities. The potential relationships between breastfeeding practices and maternal older age when giving birth, higher education, and being of Black, Asian, Mixed or other ethnic origin have been well documented by previous studies [6, 7, 11, 13, 23, 24]. In this present study, the independent associations of these sociodemographic factors may signify the importance of both theoretical and embodied knowledge of breastfeeding in the continuation of breastfeeding from 1 to 6 weeks. For instance, it has been postulated that mothers with more years of education are more likely to seek or encounter information about health, pregnancy and infant feeding, and so have more theoretical knowledge about breastfeeding, increasing their likelihood of initiating and then continuing its practise [12, 25].

In contrast, younger mothers have been found to be less likely to have theoretical knowledge of breastfeeding [26, 27], more likely to perceive themselves as unable to breastfeed, and more likely to use infant formula as they often regard the demands of breastfeeding as conflicting with their youth and thus more suited to older mothers [28, 29]. The perceptions of younger mothers may be influenced by the degree to which breastfeeding is embodied in their daily lives, including knowing of and seeing other women breastfeeding, being supported in their decision to breastfeed and forming realistic expectations about breastfeeding in their own context [30–33].

Previous UK studies have consistently shown the appearance of a ‘minority ethnic advantage’ in breastfeeding among mothers of Black, Asian, Mixed or other ethnic groups [11, 13]. They also suggest that ethnic dense communities themselves can embody the normality of breastfeeding, benefitting mothers living within them including White mothers [27, 34, 35].

These inequalities remained even after adjusting for several other factors that have been found to influence breastfeeding continuation such as receiving professional breastfeeding support [12, 25]. Most mothers in this present study reported being seen by a midwife or health visitor, most mothers (64–67% each survey year) did not report having breastfeeding problems, and only 2–6% reported having problems and not receiving professional help [18, 36]. Therefore, availability of breastfeeding support may not fully account for the observed sociodemographic inequalities. While the early introduction of breast-milk substitutes like infant formula might influence early cessation, in this study the directionality of an association with discontinuing any breastfeeding at 6 weeks is difficult to establish.

In this present study, maternal socioeconomic status was not consistently associated with breastfeeding continuation after accounting for maternal education. While having a partner may influence women’s breastfeeding practice in several ways, including through partner’s involvement in infant feeding, childcare and household labour [37, 38], other factors may be more important for the continuation of breastfeeding at 6 weeks [39]. Previous UK and USA studies found that long-term periods out of work increased the likelihood of breastfeeding exclusivity and continuation [40–43]. However, in this present study, most mothers were on maternity leave or not in paid employment at 6 weeks after birth, making it difficult to observe the influence of early return to work on breastfeeding at 6 weeks.

Lastly, this study found an increase in the prevalence of the above-mentioned subgroups of mothers who were most likely to breastfeed at 6 weeks. For example, among the mothers breastfeeding at 1 week, the proportion who had higher education increased from 21% in 1985 to 61% in 2010. Previous trend studies from the UK, USA, Norway and Spain have also concluded that increasing trends in breastfeeding initiation and continuation rates, especially after the 1970s, were driven mainly by the increasing prevalence of similar subgroups of mothers [6, 14–16]. In the UK, these changes coincided with a period of considerable increase in women’s participation in higher education and higher-skilled occupations, improvements in family-friendly policies, and shifts in family formation and the average age of childbearing [6].

### Strengths and limitations

This is the first trend analysis of sociodemographic inequalities in breastfeeding continuation in the UK over 25 years, and one of few studies to isolate the factors associated with continuation from those associated with initiation. The relatively large study populations allowed for comparisons of prevalence and ORs over time using a meta-analytic approach. They were also highly representative of the general population of mothers from which they were sampled, showing similar distributions of the sociodemographic characteristics to those seen in national data on all mothers who gave birth in each survey year in GB [36]. Therefore, the study findings likely reflect the trends and inequalities in breastfeeding at 6 weeks in the wider childbearing population. The restriction of the study populations to mothers who initiated and continued breastfeeding at 1 week allowed for the isolation of associations with breastfeeding at different time points. Few previous studies have employed a similar approach when investigating continuation.

The study has some limitations. The data analysed are based on maternal self-report, potentially resulting in reporting bias. The observed associations may be confounded by other factors not accounted for in this study. Potential biases, residual confounding and the non-collapsibility of the OR mean that differences between ORs between surveys should be interpreted cautiously [44]. The observational nature of the data also limits direct inferences of causality between factors and breastfeeding at 6 weeks. England, Wales and Scotland were not analysed separately mainly because the sample sizes of Wales and Scotland were too small for country-specific analyses. While reasonable, this approach does not assume that progress in policies and strategies, including for infant feeding [14], was identical across the three nations. The binary differentiation of ethnicity into White and Black, Asian, Mixed or other ethnicity ensured that there were enough non-White mothers to detect significant associations and was plausible given the similarly wide normalisation of breastfeeding among these groups. However, this does not imply that all women in these groups are similar with respect to their characteristics and breastfeeding needs. As with other population surveys, the response rate to the IFS declined considerably from 91% in 1985 to 51% in 2010. However, the risk of selection bias was minimised as each survey included weights that corrected for differential non-response based on maternal sociodemographic and birth-related factors. Finally, this trend study used the most recently available national data on infant feeding practices in GB and the most recent data from the 2010 survey is now ten years old. Since then, changes in the practise of breastfeeding continuation may have occurred in GB.

## Conclusion

Despite the steadily increasing trend in breastfeeding continuation from 1 week to 6 weeks from 1985 to 2010, sociodemographic inequalities in breastfeeding between subgroups of mothers remained mostly unchanged. Mothers who were younger than 30, White and had fewer years of full-time education remained especially prone to not continuing breastfeeding from 1 week to 6 weeks after birth, even after accounting for other sociodemographic, birth, health, breastfeeding experience and support factors. Population-based strategies need to pay urgent attention to the underlying psychosocial and cultural drivers in UK society that are influencing the breastfeeding decisions of these subgroups of women. The study findings also provide further evidence of the need for a more anticipatory approach to breastfeeding interventions in higher-income countries, offering targeted support from pregnancy throughout infancy for these subgroups of mothers who have characteristically remained at higher risk of early breastfeeding discontinuation even when they initiate successfully.

## Supplementary information

Supplementary Table
